# 2-(Pyrene-1-yl)-1,3-dithiane

**DOI:** 10.1107/S1600536809010320

**Published:** 2009-03-28

**Authors:** Hoong-Kun Fun, Samuel Robinson Jebas, Annada C. Maity, Nirmal K. Das, Shyamaprasad Goswami

**Affiliations:** a X-ray Crystallography Unit, School of Physics, Universiti Sains Malaysia, 11800 USM, Penang, Malaysia; b Department of Chemistry, Bengal Engineering and Science University, Shibpur, Howrah 711 103, India

## Abstract

In the title compound, C_20_H_16_S_2_, the pyrene ring is planar [maximum deviation 0.0144 (15) Å] and the dithiane ring adopts a chair conformation. The crystal packing is stabilized by C—H⋯π inter­actions. An intra­molecular C—H⋯S hydrogen bond generates an *S*(5) ring motif.

## Related literature

For thio­nation reactions, see: Goswami & Maity (2008 ▶); Goswami *et al.* (2009 ▶); Fun *et al.* (2009 ▶). For bond-length data, see: Allen *et al.* (1987 ▶). For graph-set analysis of hydrogen bonding, see: Bernstein *et al.* (1995 ▶). For ring puckering analysis, see: Cremer & Pople (1975 ▶). For the stability of the temperature controller used in the data collection, see: Cosier & Glazer (1986 ▶).
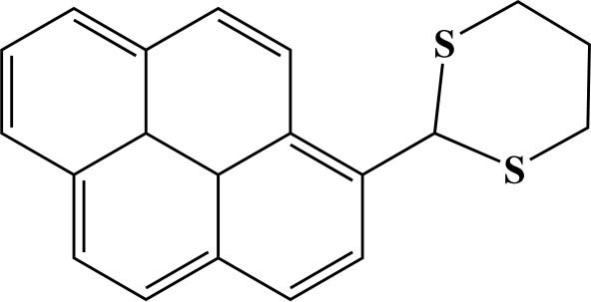


## Experimental

### 

#### Crystal data

C_20_H_16_S_2_
*M* *_r_* = 320.45
Orthorhombic, 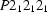

*a* = 7.1424 (1) Å
*b* = 8.6016 (1) Å
*c* = 24.5049 (2) Å
*V* = 1505.48 (3) Å^3^
*Z* = 4
Mo *K*α radiation
μ = 0.35 mm^−1^
*T* = 100 K
0.36 × 0.17 × 0.11 mm

#### Data collection

Bruker SMART APEXII CCD area-detector diffractometer
Absorption correction: multi-scan (*SADABS*; Bruker, 2005 ▶) *T*_min_ = 0.885, *T*_max_ = 0.962
29712 measured reflections
6424 independent reflections
5501 reflections with *I* > 2σ(*I*)
*R*_int_ = 0.041

#### Refinement

*R*[*F* ^2^ > 2σ(*F* ^2^)] = 0.040
*wR*(*F* ^2^) = 0.092
*S* = 1.05
6424 reflections
199 parameters
H-atom parameters constrained
Δρ_max_ = 0.48 e Å^−3^
Δρ_min_ = −0.27 e Å^−3^
Absolute structure: Flack (1983 ▶), 2662 Friedel pairs
Flack parameter: 0.03 (5)

### 

Data collection: *APEX2* (Bruker, 2005 ▶); cell refinement: *SAINT* (Bruker, 2005 ▶); data reduction: *SAINT*; program(s) used to solve structure: *SHELXTL* (Sheldrick, 2008 ▶); program(s) used to refine structure: *SHELXTL*; molecular graphics: *SHELXTL*; software used to prepare material for publication: *SHELXTL* and *PLATON* (Spek, 2009 ▶).

## Supplementary Material

Crystal structure: contains datablocks global, I. DOI: 10.1107/S1600536809010320/at2745sup1.cif

Structure factors: contains datablocks I. DOI: 10.1107/S1600536809010320/at2745Isup2.hkl

Additional supplementary materials:  crystallographic information; 3D view; checkCIF report

## Figures and Tables

**Table 1:** Hydrogen-bond geometry (Å, °)

*D*—H⋯*A*	*D*—H	H⋯*A*	*D*⋯*A*	*D*—H⋯*A*
C15—H15*A*⋯S2	0.93	2.65	3.0416 (13)	106
C9—H9*A*⋯*Cg*1^i^	0.93	2.68	3.4196 (15)	137
C4—H4*A*⋯*Cg*2^ii^	0.93	2.98	3.8073 (16)	149
C20—H20*A*⋯*Cg*3^iii^	0.97	2.78	3.5339 (15)	135

Symmetry codes: (i) 
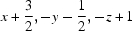
; (ii) 
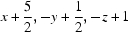
; (iii) 
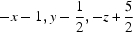
. *Cg*1 is the centroid of the C1–C6 ring, *Cg*2 is the centroid of the C1/C6–C10 ring and *Cg*3 is the centroid of the C2/C3/C13–C16 ring.

**Fig. 1.**
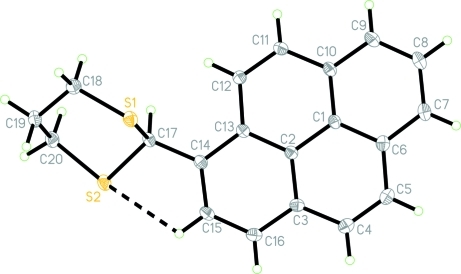
The molecular structure of the title compound, showing 50% probability displacement ellipsoids and the atom numbering scheme.

**Fig. 2.**
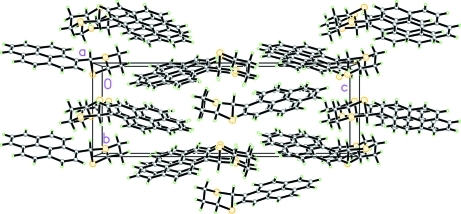
The crystal packing of the title compound, viewed along the a axis. Dashed lines indicate the hydrogen bonding.

## supplementary crystallographic information

### Comment

Thioacetal protection of carbonyl groups is of paramount importance in synthetic organic chemistry and hence the development of novel thionation reactions remains of great interest (Goswami & Maity, 2008; Fun *et al.*, 2009; Goswami *et al.*, 2009). In addition, thioacetals are also utilized as masked acyl anions or masked methylene functions in carbon-carbon bond forming reactions. Here we report the synthesis of 2-pyrene-1-yl-[1,3]dithiane from pyrene-1-aldehyde using BF_3_—Et_2_O as catalyst and its crystal structure.

The asymmetric unit of (I), (Fig. 1), consists of one molecule of the title compound. The bond lengths (Allen *et al.*, 1987) and bond angles are found to have normal values. The pyrene ring is essentially planar with the maximum deviation from planarity being 0.0144 (15)Å for atom C15. The dithiane group adopts a chair conformation with the puckering parameters Q = 0.7477 (12) Å, θ = 9.61 (10)° and φ = 66.3 (5)° (Cremer & Pople, 1975).

The crystal packing is stabilized by C—H···π interactions (Table 1). An intramolecular C—H···S hydrogen bonding generates an S(5) ring motif (Bernstein *et al.*, 1995).

### Experimental

To a stirred solution of pyrene-1-aldehyde (500 mg., 2.17 mmol) and boron trifluoride etherate (0.5 ml) in dichloromethane (50 ml) cooled at 273 K is added 1,3-propanedithiol (490 mg, 4.5 mmol) dropwise over 15 min with stirring. The mixture is stirred at room temperature for 3 h. The progress of the reaction is monitored by TLC. After completion of the reaction, NaHCO_3_ solution is added slowly and carefully to neutralize the mixture at room temperature which is then extracted with dichloromethane. The organic layer is dried (anhydrous Na_2_SO_4_) and then the solvent is removed under reduced pressure. The crude product was purified by column chromatography using silica gel with 10% ethyl acetate in pet ether as eluant to afford 2-pyrene-1-yl-[1,3]dithiane (620 mg, 89%) as a colourless crystalline solid along with other thiane derivatives.

### Refinement

H atoms were positioned geometrically [C–H = 0.93–0.98 Å] and refined using a riding model, with *U*_iso_(H) = 1.2*U*_eq_(C). 2662 Friedel pairs were used to determine the absolute configuration.

### Crystal data

**Table:**

C_20_H_16_S_2_	*F*(000) = 672
*M**_r_* = 320.45	*D*_x_ = 1.414 Mg m^−^^3^
Orthorhombic, *P*2_1_2_1_2_1_	Mo *K*α radiation, λ = 0.71073 Å
Hall symbol: P 2ac 2ab	Cell parameters from 9340 reflections
*a* = 7.1424 (1) Å	θ = 2.5–33.7°
*b* = 8.6016 (1) Å	µ = 0.35 mm^−^^1^
*c* = 24.5049 (2) Å	*T* = 100 K
*V* = 1505.48 (3) Å^3^	Block, colourless
*Z* = 4	0.36 × 0.17 × 0.11 mm

### Data collection

**Table:**

Bruker SMART APEXII CCD area-detector diffractometer	6424 independent reflections
Radiation source: fine-focus sealed tube	5501 reflections with *I* > 2σ(*I*)
graphite	*R*_int_ = 0.041
φ and ω scans	θ_max_ = 35.1°, θ_min_ = 1.7°
Absorption correction: multi-scan (*SADABS*; Bruker, 2005)	*h* = −11→11
*T*_min_ = 0.885, *T*_max_ = 0.962	*k* = −13→13
29712 measured reflections	*l* = −39→39

### Refinement

**Table:**

Refinement on *F*^2^	Secondary atom site location: difference Fourier map
Least-squares matrix: full	Hydrogen site location: inferred from neighbouring sites
*R*[*F*^2^ > 2σ(*F*^2^)] = 0.040	H-atom parameters constrained
*wR*(*F*^2^) = 0.092	*w* = 1/[σ^2^(*F*_o_^2^) + (0.0461*P*)^2^ + 0.0807*P*] where *P* = (*F*_o_^2^ + 2*F*_c_^2^)/3
*S* = 1.05	(Δ/σ)_max_ < 0.004
6424 reflections	Δρ_max_ = 0.48 e Å^−^^3^
199 parameters	Δρ_min_ = −0.27 e Å^−^^3^
0 restraints	Absolute structure: Flack (1983), 2662 Friedel pairs
Primary atom site location: structure-invariant direct methods	Flack parameter: 0.03 (5)

### Special details

**Table:**

Experimental. The crystal was placed in the cold stream of an Oxford Cyrosystems Cobra open-flow nitrogen cryostat (Cosier & Glazer, 1986) operating at 100.0 (1) K.
Geometry. All e.s.d.'s (except the e.s.d. in the dihedral angle between two l.s. planes) are estimated using the full covariance matrix. The cell e.s.d.'s are taken into account individually in the estimation of e.s.d.'s in distances, angles and torsion angles; correlations between e.s.d.'s in cell parameters are only used when they are defined by crystal symmetry. An approximate (isotropic) treatment of cell e.s.d.'s is used for estimating e.s.d.'s involving l.s. planes.
Refinement. Refinement of *F*^2^ against ALL reflections. The weighted *R*-factor *wR* and goodness of fit *S* are based on *F*^2^, conventional *R*-factors *R* are based on *F*, with *F* set to zero for negative *F*^2^. The threshold expression of *F*^2^ > σ(*F*^2^) is used only for calculating *R*-factors(gt) *etc*. and is not relevant to the choice of reflections for refinement. *R*-factors based on *F*^2^ are statistically about twice as large as those based on *F*, and *R*- factors based on ALL data will be even larger.

### Fractional atomic coordinates and isotropic or equivalent isotropic displacement parameters (Å^2^)

**Table:**

	*x*	*y*	*z*	*U*_iso_*/*U*_eq_	
S1	0.38288 (5)	0.10233 (4)	0.977454 (13)	0.01668 (8)	
S2	0.68513 (5)	−0.07534 (4)	1.037145 (12)	0.01665 (8)	
C1	0.6709 (2)	−0.12016 (15)	0.77433 (5)	0.0134 (2)	
C2	0.73272 (19)	−0.07300 (16)	0.82744 (5)	0.0131 (2)	
C3	0.9112 (2)	−0.00108 (16)	0.83308 (5)	0.0140 (2)	
C4	1.0265 (2)	0.01927 (17)	0.78565 (6)	0.0173 (3)	
H4A	1.1443	0.0640	0.7894	0.021*	
C5	0.9675 (2)	−0.02513 (17)	0.73559 (6)	0.0177 (3)	
H5A	1.0456	−0.0107	0.7057	0.021*	
C6	0.7869 (2)	−0.09392 (16)	0.72787 (5)	0.0156 (3)	
C7	0.7187 (2)	−0.13545 (17)	0.67611 (5)	0.0180 (3)	
H7A	0.7932	−0.1193	0.6455	0.022*	
C8	0.5425 (2)	−0.19998 (17)	0.66994 (6)	0.0190 (3)	
H8A	0.4998	−0.2264	0.6353	0.023*	
C9	0.4287 (2)	−0.22556 (16)	0.71502 (5)	0.0180 (3)	
H9A	0.3102	−0.2682	0.7103	0.022*	
C10	0.4912 (2)	−0.18750 (16)	0.76761 (5)	0.0145 (2)	
C11	0.3773 (2)	−0.21097 (16)	0.81490 (5)	0.0162 (3)	
H11A	0.2612	−0.2588	0.8110	0.019*	
C12	0.4341 (2)	−0.16537 (17)	0.86520 (5)	0.0155 (3)	
H12A	0.3555	−0.1810	0.8950	0.019*	
C13	0.61341 (19)	−0.09331 (15)	0.87345 (5)	0.0129 (2)	
C14	0.6739 (2)	−0.03780 (15)	0.92506 (5)	0.0139 (2)	
C15	0.8468 (2)	0.03716 (16)	0.92932 (5)	0.0159 (3)	
H15A	0.8838	0.0771	0.9629	0.019*	
C16	0.9649 (2)	0.05354 (16)	0.88468 (5)	0.0164 (3)	
H16A	1.0808	0.1012	0.8890	0.020*	
C17	0.54924 (19)	−0.05773 (15)	0.97455 (5)	0.0142 (2)	
H17A	0.4780	−0.1542	0.9698	0.017*	
C18	0.2364 (2)	0.03068 (18)	1.03263 (5)	0.0201 (3)	
H18A	0.1366	0.1049	1.0391	0.024*	
H18B	0.1791	−0.0663	1.0213	0.024*	
C19	0.3411 (2)	0.00340 (18)	1.08623 (5)	0.0184 (3)	
H19A	0.3993	0.1001	1.0975	0.022*	
H19B	0.2516	−0.0259	1.1142	0.022*	
C20	0.4911 (2)	−0.12196 (17)	1.08241 (5)	0.0174 (3)	
H20A	0.4331	−0.2175	1.0699	0.021*	
H20B	0.5404	−0.1410	1.1187	0.021*	

### Atomic displacement parameters (Å^2^)

**Table:**

	*U*^11^	*U*^22^	*U*^33^	*U*^12^	*U*^13^	*U*^23^
S1	0.01679 (16)	0.01854 (16)	0.01470 (14)	0.00372 (13)	−0.00236 (11)	0.00034 (12)
S2	0.01684 (15)	0.02099 (17)	0.01214 (13)	0.00328 (14)	−0.00233 (11)	0.00104 (12)
C1	0.0165 (6)	0.0116 (5)	0.0120 (5)	0.0012 (5)	−0.0008 (4)	−0.0008 (4)
C2	0.0142 (6)	0.0121 (6)	0.0131 (5)	0.0004 (5)	−0.0012 (4)	0.0010 (4)
C3	0.0131 (6)	0.0128 (6)	0.0160 (5)	0.0010 (5)	−0.0021 (5)	0.0015 (4)
C4	0.0148 (7)	0.0169 (6)	0.0203 (6)	0.0002 (5)	0.0011 (5)	0.0020 (5)
C5	0.0171 (7)	0.0185 (7)	0.0177 (6)	0.0019 (5)	0.0038 (5)	0.0024 (5)
C6	0.0174 (6)	0.0145 (6)	0.0148 (5)	0.0021 (5)	0.0004 (4)	0.0012 (5)
C7	0.0236 (7)	0.0177 (7)	0.0128 (6)	0.0025 (5)	0.0003 (5)	0.0001 (5)
C8	0.0256 (7)	0.0182 (7)	0.0133 (6)	0.0021 (6)	−0.0034 (5)	−0.0019 (5)
C9	0.0216 (7)	0.0158 (6)	0.0166 (6)	−0.0008 (5)	−0.0035 (5)	−0.0020 (5)
C10	0.0178 (6)	0.0124 (6)	0.0134 (5)	0.0001 (5)	−0.0017 (5)	−0.0019 (4)
C11	0.0159 (6)	0.0169 (6)	0.0159 (6)	−0.0033 (5)	−0.0015 (5)	−0.0010 (5)
C12	0.0159 (6)	0.0164 (6)	0.0141 (5)	−0.0028 (5)	0.0002 (5)	0.0007 (5)
C13	0.0153 (6)	0.0103 (5)	0.0130 (5)	0.0004 (5)	−0.0015 (4)	0.0001 (4)
C14	0.0151 (6)	0.0133 (6)	0.0133 (5)	0.0012 (5)	−0.0021 (5)	0.0003 (4)
C15	0.0181 (7)	0.0157 (6)	0.0138 (5)	−0.0010 (5)	−0.0043 (5)	−0.0001 (5)
C16	0.0147 (6)	0.0160 (6)	0.0186 (6)	−0.0016 (5)	−0.0027 (5)	0.0016 (5)
C17	0.0163 (6)	0.0148 (6)	0.0115 (5)	0.0014 (5)	−0.0024 (4)	−0.0008 (4)
C18	0.0154 (6)	0.0269 (7)	0.0179 (6)	0.0021 (6)	−0.0001 (5)	−0.0018 (5)
C19	0.0212 (7)	0.0212 (7)	0.0128 (5)	0.0008 (6)	0.0005 (5)	−0.0010 (5)
C20	0.0207 (7)	0.0179 (7)	0.0136 (5)	−0.0005 (6)	0.0001 (5)	0.0018 (5)

### Geometric parameters (Å, °)

**Table:**

S1—C18	1.8173 (15)	C9—H9A	0.9300
S1—C17	1.8200 (14)	C10—C11	1.4301 (19)
S2—C20	1.8200 (15)	C11—C12	1.3557 (18)
S2—C17	1.8214 (13)	C11—H11A	0.9300
C1—C10	1.418 (2)	C12—C13	1.4373 (19)
C1—C6	1.4260 (18)	C12—H12A	0.9300
C1—C2	1.4328 (17)	C13—C14	1.4191 (17)
C2—C3	1.4239 (19)	C14—C15	1.397 (2)
C2—C13	1.4242 (17)	C14—C17	1.5144 (18)
C3—C16	1.4024 (18)	C15—C16	1.3884 (19)
C3—C4	1.4349 (19)	C15—H15A	0.9300
C4—C5	1.3521 (19)	C16—H16A	0.9300
C4—H4A	0.9300	C17—H17A	0.9800
C5—C6	1.432 (2)	C18—C19	1.5298 (19)
C5—H5A	0.9300	C18—H18A	0.9700
C6—C7	1.4047 (18)	C18—H18B	0.9700
C7—C8	1.384 (2)	C19—C20	1.523 (2)
C7—H7A	0.9300	C19—H19A	0.9700
C8—C9	1.389 (2)	C19—H19B	0.9700
C8—H8A	0.9300	C20—H20A	0.9700
C9—C10	1.4025 (18)	C20—H20B	0.9700
			
C18—S1—C17	98.54 (7)	C13—C12—H12A	119.4
C20—S2—C17	97.23 (6)	C14—C13—C2	118.82 (12)
C10—C1—C6	119.86 (11)	C14—C13—C12	122.81 (12)
C10—C1—C2	120.01 (12)	C2—C13—C12	118.34 (11)
C6—C1—C2	120.09 (12)	C15—C14—C13	119.42 (12)
C3—C2—C13	120.81 (11)	C15—C14—C17	120.80 (11)
C3—C2—C1	119.16 (12)	C13—C14—C17	119.77 (12)
C13—C2—C1	120.00 (12)	C16—C15—C14	121.66 (12)
C16—C3—C2	118.55 (12)	C16—C15—H15A	119.2
C16—C3—C4	122.18 (13)	C14—C15—H15A	119.2
C2—C3—C4	119.21 (12)	C15—C16—C3	120.69 (13)
C5—C4—C3	121.43 (14)	C15—C16—H16A	119.7
C5—C4—H4A	119.3	C3—C16—H16A	119.7
C3—C4—H4A	119.3	C14—C17—S1	109.24 (9)
C4—C5—C6	121.17 (13)	C14—C17—S2	111.75 (9)
C4—C5—H5A	119.4	S1—C17—S2	112.20 (7)
C6—C5—H5A	119.4	C14—C17—H17A	107.8
C7—C6—C1	118.64 (13)	S1—C17—H17A	107.8
C7—C6—C5	122.46 (12)	S2—C17—H17A	107.8
C1—C6—C5	118.89 (11)	C19—C18—S1	114.16 (10)
C8—C7—C6	121.05 (13)	C19—C18—H18A	108.7
C8—C7—H7A	119.5	S1—C18—H18A	108.7
C6—C7—H7A	119.5	C19—C18—H18B	108.7
C7—C8—C9	120.58 (13)	S1—C18—H18B	108.7
C7—C8—H8A	119.7	H18A—C18—H18B	107.6
C9—C8—H8A	119.7	C20—C19—C18	113.57 (11)
C8—C9—C10	120.51 (14)	C20—C19—H19A	108.9
C8—C9—H9A	119.7	C18—C19—H19A	108.9
C10—C9—H9A	119.7	C20—C19—H19B	108.9
C9—C10—C1	119.35 (13)	C18—C19—H19B	108.9
C9—C10—C11	122.04 (13)	H19A—C19—H19B	107.7
C1—C10—C11	118.60 (11)	C19—C20—S2	114.65 (10)
C12—C11—C10	121.71 (13)	C19—C20—H20A	108.6
C12—C11—H11A	119.1	S2—C20—H20A	108.6
C10—C11—H11A	119.1	C19—C20—H20B	108.6
C11—C12—C13	121.29 (13)	S2—C20—H20B	108.6
C11—C12—H12A	119.4	H20A—C20—H20B	107.6
			
C10—C1—C2—C3	−178.01 (12)	C10—C11—C12—C13	−1.0 (2)
C6—C1—C2—C3	−0.47 (19)	C3—C2—C13—C14	1.21 (19)
C10—C1—C2—C13	−0.20 (19)	C1—C2—C13—C14	−176.56 (12)
C6—C1—C2—C13	177.34 (12)	C3—C2—C13—C12	179.43 (12)
C13—C2—C3—C16	−1.84 (19)	C1—C2—C13—C12	1.66 (19)
C1—C2—C3—C16	175.95 (13)	C11—C12—C13—C14	177.07 (13)
C13—C2—C3—C4	−179.16 (13)	C11—C12—C13—C2	−1.1 (2)
C1—C2—C3—C4	−1.37 (19)	C2—C13—C14—C15	0.97 (19)
C16—C3—C4—C5	−175.68 (14)	C12—C13—C14—C15	−177.17 (13)
C2—C3—C4—C5	1.5 (2)	C2—C13—C14—C17	179.86 (12)
C3—C4—C5—C6	0.2 (2)	C12—C13—C14—C17	1.7 (2)
C10—C1—C6—C7	0.25 (19)	C13—C14—C15—C16	−2.6 (2)
C2—C1—C6—C7	−177.30 (13)	C17—C14—C15—C16	178.56 (12)
C10—C1—C6—C5	179.72 (13)	C14—C15—C16—C3	1.9 (2)
C2—C1—C6—C5	2.17 (19)	C2—C3—C16—C15	0.3 (2)
C4—C5—C6—C7	177.38 (14)	C4—C3—C16—C15	177.52 (13)
C4—C5—C6—C1	−2.1 (2)	C15—C14—C17—S1	93.99 (13)
C1—C6—C7—C8	0.3 (2)	C13—C14—C17—S1	−84.88 (13)
C5—C6—C7—C8	−179.18 (13)	C15—C14—C17—S2	−30.77 (16)
C6—C7—C8—C9	−0.2 (2)	C13—C14—C17—S2	150.36 (10)
C7—C8—C9—C10	−0.5 (2)	C18—S1—C17—C14	171.69 (9)
C8—C9—C10—C1	1.0 (2)	C18—S1—C17—S2	−63.82 (9)
C8—C9—C10—C11	179.45 (13)	C20—S2—C17—C14	−172.85 (9)
C6—C1—C10—C9	−0.88 (19)	C20—S2—C17—S1	64.05 (8)
C2—C1—C10—C9	176.67 (13)	C17—S1—C18—C19	59.16 (12)
C6—C1—C10—C11	−179.38 (13)	S1—C18—C19—C20	−63.42 (15)
C2—C1—C10—C11	−1.83 (19)	C18—C19—C20—S2	65.04 (15)
C9—C10—C11—C12	−175.99 (14)	C17—S2—C20—C19	−61.20 (11)
C1—C10—C11—C12	2.5 (2)		

### Hydrogen-bond geometry (Å, °)

**Table:**

*D*—H···*A*	*D*—H	H···*A*	*D*···*A*	*D*—H···*A*
C15—H15A···S2	0.93	2.65	3.0416 (13)	106
C9—H9A···Cg1^i^	0.93	2.68	3.4196 (15)	137
C4—H4A···Cg2^ii^	0.93	2.98	3.8073 (16)	149
C20—H20A···Cg3^iii^	0.97	2.78	3.5339 (15)	135

Symmetry codes: (i) *x*+3/2, −*y*−1/2, −*z*+1; (ii) *x*+5/2, −*y*+1/2, −*z*+1; (iii) −*x*−1, *y*−1/2, −*z*+5/2.
